# Machine Learning Models for Identifying Factors Associated With Workplace Violence Among Emergency Nurses: A Comparative Study

**DOI:** 10.1155/emmi/9275645

**Published:** 2026-08-19

**Authors:** Lin Lan, Min Dai, Yilong Chen, Hao Zhang, Luying Zhong, Shan Tang, Xiaoli Chen

**Affiliations:** ^1^ Emergency Department of West China Hospital, Sichuan University/West China School of Nursing, Sichuan University, Chengdu 610041, China, scu.edu.cn; ^2^ Institute of Disaster Medicine, Sichuan University, Chengdu 610041, China, scu.edu.cn; ^3^ West China Biomedical Big Data Center, West China Hospital, Sichuan University, Chengdu 610041, China, scu.edu.cn; ^4^ Med-X Center for Informatics, Sichuan University, Chengdu 610041, China, scu.edu.cn; ^5^ Nursing Department, First Hospital of Shanxi Medical University, Taiyuan 030001, Shanxi, China, sxmu.edu.cn

**Keywords:** associated factors, classification model, emergency nurses, machine learning, workplace violence

## Abstract

**Objective:**

To develop and compare three machine learning models for identifying factors associated with workplace violence (WPV) among emergency department nurses.

**Methods:**

A total of 1540 emergency department nurses from various regions of China were examined between December 2023 and January 2024. Data were collected using scales measuring WPV, work–family conflict, occupational stress, occupational burnout, nursing practice environment, and self‐rated sleep quality. Three analytical models (logistic regression, decision tree, and random forest) were developed and compared to classify nurses with and without WPV experiences among emergency department nurses. Model performance was evaluated using sensitivity, specificity, PPV, NPV, F1‐score, balanced accuracy, and area under the receiver operating characteristic curve (AUC).

**Results:**

Among the 1540 nurses, 1309 individuals (85.0%) had experienced WPV in the past year. All three models indicated that work–family conflict, occupational burnout, and occupational stress were significantly associated with WPV experiences among emergency department nurses (*p* < 0.05). In the random forest and decision tree models, sleep disorders and the nursing practice environment were also identified as significant associated factors. The accuracy of the logistic regression, decision tree, and random forest models was 0.829, 0.851, and 0.859; the specificity was 0.667, 0.605, and 0.593; the sensitivity was 0.864, 0.903, and 0.916; and the F1 score was 0.893, 0.909, and 0.915, respectively, with AUC values of 0.832 (95% CI: 0.781–0.883), 0.768 (95% CI: 0.711–0.826), and 0.834 (95% CI: 0.783–0.885).

**Conclusions:**

Work–family conflict, burnout, occupational stress, the nursing practice environment, and sleep disorders were consistently associated with WPV experiences among emergency department nurses. Random forest achieved the highest sensitivity, F1‐score, and overall accuracy, whereas logistic regression showed the highest specificity and balanced accuracy. Logistic regression and random forest demonstrated similar discrimination.

## 1. Introduction

According to the World Health Organization’s definition, workplace violence (WPV) refers to “incidents where staff are abused, threatened, or assaulted in circumstances related to their work, involving an explicit or implicit challenge to their safety, well‐being, or health” [1]. This definition encompasses not only physical assaults but also psychological forms of violence such as verbal abuse, threats, bullying, and sexual harassment [2]. WPV represents a global challenge in the healthcare sector, with particularly high prevalence in emergency departments. Studies indicate that 19.3%–36.4% of hospital staff have experienced physical violence, with emergency nurses being the most affected group, reporting prevalence rates as high as 79.39%–92.9% [3]. The impact of WPV on nurses’ physical and mental health is extensive and profound, potentially triggering anxiety, depression, post‐traumatic stress disorder, and psychological distress [4]. It significantly increases their intention to leave the profession [5]. Furthermore, WPV may elevate the risk of medication errors and other adverse events [6]. While healthcare institutions have gradually recognized the severity of WPV and implemented corresponding management measures, such as increasing security personnel, improving medical procedures and environments [7], and conducting antiviolence and patient communication training [8], most strategies remain reactive and lack specificity, resulting in limited effectiveness in preventing violent incidents [9]. Consequently, identifying nurses and work units with characteristics associated with WPV may facilitate targeted prevention strategies in reducing WPV against emergency nurses, holding significant implications for ensuring patient safety and enhancing the physical and mental well‐being of healthcare professionals.

The occurrence of WPV results from a complex interplay of multifaceted factors, encompassing hospital management elements, nurses’ individual characteristics, and factors related to patients and their families. Previous research has extensively explored the negative impacts of violent incidents on nurses’ physical and mental health and career development and has made some progress in identifying associated factors such as focusing on the personality traits of patients and their families, basic nurse characteristics (age, gender, education level, and years of experience), and the nature of healthcare institutions [10]. However, these factors are largely static and difficult to modify, and existing analyses have mostly been limited to examining individual factors in isolation, lacking a systematic integration of risk elements and an integrated analytical framework. In recent years, there has been growing attention on the relationship between nurses’ psychological characteristics such as job burnout, stress, and work–family conflict and WPV. Nevertheless, existing studies have paid insufficient attention to high‐risk groups such as emergency nurses, have rarely examined the role of nurses’ individual psychological traits associated with WPV experiences, and have not yet examined these psychological variables within an integrated analytical framework [11]. Burnout and stress have been reported to be associated with WPV among nurses. In terms of actionable practice, we emphasize that these psychological factors should be viewed as important indicators of psychosocial and organizational strain.

Machine learning technology, with its capability to mine complex nonlinear patterns from high‐dimensional data, has demonstrated exceptional performance in healthcare scenarios such as disease diagnosis and prognosis assessment [12]. Introducing machine learning models into examining WPV‐associated characteristics enables the identification of nurses reporting characteristics associated with WPV experiences and adverse psychosocial conditions, thereby providing data‐driven decision support for managers. Some studies have attempted to apply machine learning models; for instance, a Canadian [13] cross‐sectional survey of 4066 nurses used machine learning to identify factors associated with WPV, identifying the lack of security measures and work overload as associated factors. A Chinese study [14] of 5663 nurses utilizing the Light Gradient Boosting Machine model showed that patient complaints, nurses’ psychological demands, and professional identity were associated factors of WPV. However, these studies mainly employed a single algorithm, which led to differences in the research results. Different machine learning models vary in principles and performance, making a direct comparison of results challenging. For example, the logistic regression model operates as a rigid formula assuming linear additive relationships among factors. Decision trees [15] function as clear flowcharts with intuitively understandable rules. Random forest [16] by constructing numerous decision trees effectively mitigates overfitting issues inherent in single trees and excels at capturing complex interactions among factors. Consequently, which model demonstrates superior performance in identifying nurses reporting WPV experiences and, more importantly, which offers the best balance between evaluable accuracy and interpretability for practical application in clinical settings remain unclear.

Therefore, to address this gap, the present study aims to conduct a comparative analysis of three representative machine learning models, logistic regression, decision tree, and random forest, for examining WPV experiences among emergency nurses.

The study was guided by a conceptual framework in which organizational conditions (work environment and workload‐related stressors) may influence nurses’ psychosocial responses (occupational stress, burnout, and work–family conflict), which in turn may be associated with WPV experiences. Sleep disturbance was considered an additional indicator of occupational strain. Based on this framework, multiple psychosocial and occupational variables were simultaneously examined using machine learning approaches.

### 1.1. Objective

This study aimed to systematically examine the associations between psychological, occupational, and behavioral characteristics and WPV among emergency nurses. By developing and comparing three machine learning models (logistic regression, decision tree, and random forest), we sought to identify factors most strongly associated with WPV experiences and evaluate the relative performance of different analytical approaches. The findings may contribute to the development of organizational strategies aimed at reducing WPV.

### 1.2. Design

A cross‐sectional survey design was used in this study to examine health status, work characteristics, and family‐related factors among emergency nurses.

### 1.3. Settings

A nationwide cross‐sectional survey was conducted using nonprobability convenience sampling through hospital‐based and professional nursing networks.

### 1.4. Participants

Emergency nursing staff who met the following criteria were included in the study: (1) registered nurses working in emergency departments; (2) age ≥ 18 years; and (3) emergency department work experience ≥ 1 year. The exclusion criteria were as follows: (1) severe psychiatric conditions or current psychotropic medication use that could interfere with independent questionnaire completion; (2) undergoing advanced training; and (3) nurses on sick leave, maternity leave, or breastfeeding leave (for ≥ 1 month). The minimum required sample size was calculated based on the following parameters: a two‐sided significance level (α) of 0.05, a statistical power (1‐β) of 80%, and a standard deviation of anxiety scores (σ) of approximately 0.93, derived from our pre‐survey data. This calculation yielded a minimum required sample size of 1,330 participants. The present study was a secondary analysis of an existing national survey dataset.

## 2. Methods

The survey comprehensively evaluated a range of factors pertinent to emergency nurses, including somatic symptoms, sleep quality, WPV, occupational burnout, work environment, work–family role conflict, and occupational stress. As of now, five articles have been published based on the data collected in this survey. The five studies all utilized this dataset, but each study focused on a different research direction and employed traditional statistical methods (regression analysis, mediation analysis, and latent profile analysis). The present study is the first to apply and compare multiple machine learning models for examining factors associated with WPV among emergency nurses.

This study is reported in accordance with the STROBE guidelines for observational studies. This survey encompassed three primary components: sociodemographic information, WPV and work status, and nurse health status.

Sociodemographic Information: This section collected data on gender, age, educational level, years of work experience, night shift frequency, income level, marital status, and childbearing status. All instruments used in this study were Chinese versions that had demonstrated satisfactory validity and reliability in previous studies involving Chinese nurses [17].

WPV: Four WPV items were adapted from the 2005 National Survey of the Work and Health of Nurses (Statistics Canada, 2006) and the study by Hesketh et al. [18]. Participants were asked to indicate the frequency with which they had experienced (a) emotional abuse from patients and/or families, (b) emotional abuse from employers and/or colleagues, (c) physical abuse from patients and/or families, and (d) physical abuse from employers and/or colleagues in their primary workplace over the past year (0 = Never, 6 = Daily). A higher mean score indicates a greater frequency of WPV experiences. Cronbach’s *α* for the scale in the present study was 0.71.

Work–Family Behavioral Role Conflict Scale: The Work–Family Behavioral Role Conflict Scale (WFBRC‐S) was applied in its Chinese version, which was adapted by Zhang et al. [19] from the original instrument developed by Clark et al. [20]. This scale assesses the extent to which role pressures from work and family domains are mutually incompatible, specifically the degree to which participation in one role (e.g., work) interferes with participation in the other role (e.g., family). The scale distinguishes two dimensions of behavioral role conflict: work‐to‐family and family‐to‐work conflict, with a total of 19 items. Responses are recorded on a 5‐point frequency scale ranging from 1 (“Never”) to 5 (“Very Frequently”), where higher scores reflect a higher frequency of behavioral role conflict experienced by the employee. In the current study, the scale showed excellent internal consistency, with Cronbach’s *α* of 0.95.

Occupational Stress Questionnaire: The questionnaire developed by the German professor Johannes Siegrist [21] based on the Effort‐Reward Imbalance (ERI) model was used to assess occupational stress factors. Comparative analyses of data from five European countries (e.g., Germany, the United Kingdom, Sweden) have demonstrated that this instrument provides a psychometrically sound measure for the comparative assessment of work‐related stress grounded in sociological theory. The questionnaire consists of 6 items for “Effort,” 11 items for “Reward,” and 5 items for “Overcommitment.” All items are rated on a 5‐point Likert scale, with scores ranging from 1 to 5. Higher total scores indicate a higher level of occupational stress.

Occupational Burnout: The Maslach Burnout Inventory‐General Survey (MBI‐GS), in its Chinese adaptation, was employed to evaluate burnout levels among participants. Initially developed by Maslach and Leiter [22], the instrument was later localized by Zhu et al. [23]. This version is recognized for its broad applicability across diverse professional fields and has demonstrated robust cross‐cultural utility. Its reliability and validity have been substantiated in domestic studies. Comprising 16 items, the MBI‐GS uses a 7‐point Likert scale ranging from 0 to 6. The measure is organized into three subscales: Emotional Exhaustion, Depersonalization, and Personal Accomplishment. Scores on the first two subscales are positively keyed, where higher values correspond to greater burnout. In contrast, the Personal Accomplishment subscale is reverse‐scored, with lower ratings reflecting higher burnout. An overall burnout index is computed using the formula [0.4 × Emotional Exhaustion + 0.3 × Depersonalization + 0.3 × (6 − Personal Accomplishment)]. Based on this composite score, burnout levels are classified as follows: No Burnout (< 1.5), Suspected Burnout (1.5 to < 3.5), and Confirmed Burnout (≥ 3.5). In the present study, Cronbach’s *α* coefficient was 0.860.

Nursing Practice Work Environment: The Nursing Work Environment Scale, developed by Ye [24], was used to assess the practice environment. It comprises 26 items across seven dimensions: Career Development, Leadership and Management, Nurse–Physician Relations, Recognition Atmosphere, Professional Autonomy, Basic Support, and Adequate Staffing. Responses are recorded on a 6‐point Likert scale, ranging from “Strongly Disagree” (1 point) to “Strongly Agree” (6 points). The total score ranges from 26 to 156, with higher scores indicating greater satisfaction with the work environment. In this study, the overall Cronbach’s *α* for the scale was 0.946, while the coefficients for the seven dimensions ranged from 0.799 to 0.896.

Sleep Disorder Assessment: Sleep disturbances were measured with the Self‐Administered Sleep Questionnaire (SSQ) [25], which includes three items assessing distinct sleep symptoms: sleep latency, difficulty maintaining sleep, and early morning awakening. A participant was classified as having a sleep disorder if one or more of the following conditions were met: taking longer than 30 min to fall asleep, reporting difficulty maintaining sleep “almost every night,” or experiencing early morning awakening “almost every night.” Higher total scores on the SSQ reflect greater severity of sleep problems. In the current study, Cronbach’s *α* coefficient was 0.786.

### 2.1. Data Collection

An electronic questionnaire containing an informed consent form was developed using Wenjuanxing, a widely used online survey platform in China. Access was restricted to one submission per IP address to reduce duplicate responses. This study used a nationwide, multicenter convenience sampling approach. The questionnaire was disseminated through nursing scholars attending a national conference and designated coordinators at participating hospitals. The coordinators received standardized training regarding the study purpose, eligibility criteria, key concepts, and questionnaire instructions to ensure consistent communication with potential participants.

During the data collection period, the questionnaire link, presented as a QR code, was distributed to potentially eligible nurses through WeChat. Before accessing the questionnaire, potential participants were informed of the study purpose and were required to read the electronic informed consent form and indicate their agreement to participate. Participants were advised to contact the coordinator at their hospital if they had questions.

### 2.2. Statistical Analysis and Machine Learning Modeling

Analyses were performed using SPSS 26.0 and R 4.2.3, with the rpart and randomForest packages. Continuous data with normal distribution are presented as mean standard deviation and compared via an independent‐samples t‐test. Categorical data are expressed as frequencies and percentages and analyzed using the chi‐square or rank‐sum test. Variable associations were evaluated using Pearson or point‐biserial correlation coefficients.

Univariate analyses were conducted to describe the crude associations between participant characteristics. Candidate predictors were selected based on the conceptual framework, previous studies, and their occupational, physiological, or psychological relevance. The same candidate predictor set was used in the logistic regression, decision tree, and random forest models.

The dataset was randomly divided into a training set (70%, *n* = 1078) and a testing set (30%, *n* = 462). Logistic regression, decision tree, and random forest models were developed using the training set. The decision tree model was fitted using the rpart package with its default settings, including Gini impurity as the splitting criterion, cost‐complexity pruning with cp = 0.01, minsplit = 20, minbucket = 7, and maxdepth = 30. The random forest model was fitted using the random forest package with default settings, including ntree = 500, mtry = 3, Gini impurity as the split criterion, nodesize = 1, and bootstrap sampling with replacement. The random seed used for data splitting and model construction was 18,363. Due to the high prevalence of WPV (84.93%) and data imbalance, model performance was evaluated on the test set using AUC, sensitivity, specificity, PPV, NPV, F1‐score, and balanced accuracy. An additional 10‐fold cross‐validation was performed within the training set to assess model stability and optimize the decision tree and random forest models.

## 3. Results

### 3.1. General Characteristics of the Participants and Incidence of WPV

**FIGURE 1:**
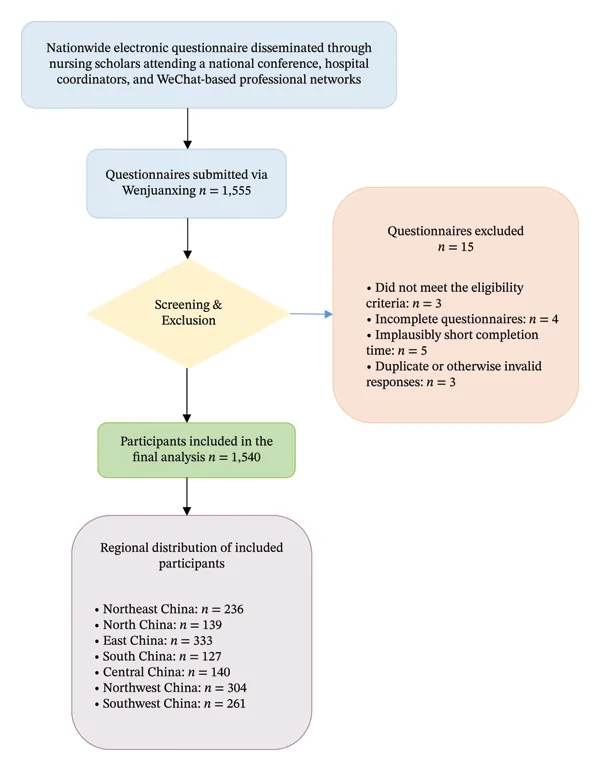
Participant flow diagram.

A total of 1555 questionnaires were collected, with 1540 being valid. The participant flow diagram is shown in Figure 1. The majority of participants were female nurses (78.6%). Overall, 85.0% (*n* = 1309) had experienced WPV in the past year. Regarding the source of violence, 84.2% (*n* = 1297) reported WPV from patients or their families, and 53.2% (*n* = 819) reported WPV from colleagues or supervisors. Regarding the type of violence, 61.3% (*n* = 943) reported physical violence and 84.4% (*n* = 1299) reported psychological violence, shown in Table 1. The mean WPV score among emergency department nurses was 2.2 ± 1.97.

**TABLE 1:** Distribution of workplace violence by type and source among participants (*N* = 1540).

Source of workplace violence	Physical violence, *n* (%)	Psychological violence, *n* (%)
Any source	943 (61.2)	1299 (84.4)
Patients or patients’ family members	918 (59.6)	1287 (83.6)
Supervisors or colleagues	406 (26.4)	712 (46.2)

The dataset was randomly divided into a training set (70%, *n* = 1078) and a testing set (30%, *n* = 462). Tenfold cross‐validation was performed within the training set as a sensitivity analysis for model tuning and stability assessment. Age and years of work experience were compared between the two sets using independent‐samples *t*‐tests, while gender and WPV prevalence were compared using chi‐square tests. No significant differences were found in these variables (all *p* > 0.05), indicating comparable baseline characteristics between the two sets.

Training Set: The training set comprised 1078 participants. Their ages ranged from 21 to 58 years (mean = 32.45 ± 6.84). Among them, 851 (79.68%) were female, 951 (89.04%) held a bachelor’s degree or higher, and 619 (57.96%) held junior professional titles. Regarding work experience, 338 (31.65%) had less than 5 years of service. In terms of work patterns, 553 (51.78%) reported weekly working hours of 41–48 h, 951 (89.04%) worked night shifts, and among those, 465 (43.54%) reported more than 8 night shifts per month. A total of 928 participants (86.89%) had experienced WPV. Concerning income, 502 (47%) reported a monthly income greater than ¥10,000 (approximately $1455 USD). Regarding training, 632 (59.18%) had received training related to WPV prevention and management. Testing Set: The testing set included 462 participants, aged 20–58 years (mean = 31.73 ± 6.68). This group consisted of 360 (77.92%) females, 403 (87.23%) with a bachelor’s degree or higher, and 288 (62.34%) with junior professional titles. A total of 158 participants (34.20%) had less than 5 years of work experience. Weekly working hours of 41–48 h were reported by 227 individuals (49.13%). Night shifts were worked by 408 (88.31%), with 194 (41.99%) working more than 8 night shifts per month. The prevalence of WPV in this set was 82.47% (*n* = 381). In terms of socioeconomic factors, 214 (46.32%) had a monthly income exceeding ¥10,000 (approximately $1455 USD), and 266 (57.58%) had received WPV prevention and management training.

### 3.2. Univariate Analysis of WPV Among Emergency Department Nurses

Using the occurrence of WPV as the dependent variable, univariate analysis was performed. The results revealed statistically significant differences (*p* < 0.05) in the distribution of WPV across the physiological and psychological work behavior characteristics. Detailed results are presented in Supporting Table S1.

### 3.3. Development of Three Classification Models for WPV Among Emergency Department Nurses

Logistic regression analysis: Binary logistic regression was used to examine factors associated with WPV among nurses. WPV was coded as 0 = No and 1 = Yes. Candidate predictors were selected a priori based on the conceptual framework, previous studies, and their occupational, physiological, or psychological relevance. The same predictor set was used in both the logistic regression and machine learning models. Variable coding is presented in Supporting Table S2. All variance inflation factor values were below 5, indicating no substantial multicollinearity (Table 2).

**TABLE 2:** Correlation analysis of continuous variables and WPV.

Variable	WPV	M ± SD	*t*	*p*
Occupational burnout	No	7.25 ± 5.122	0.229	< 0.001
	Yes	11.95 ± 5.731		

Work environment	No	133.85 ± 26.558	5.229	< 0.001
	Yes	114.67 ± 23.08		

Work‐to‐family conflict	No	12.39 ± 6.392	13.229	< 0.001
	Yes	19.31 ± 6.426		

As shown in Table 3, work–family conflict, occupational stress, burnout, and years of work experience were significantly associated with WPV among emergency department nurses (all *p* < 0.05).

**TABLE 3:** Results of logistic regression analysis on WPV against nurses in the emergency department.

Variable	*β*	SE	OR	95% CI		*p*
				Lower	Upper	
Constant work‐to‐family conflict	0.029	0.010	1.029	1.009	1.051	0.003
Occupational stress	0.031	0.011	1.031	1.009	1.054	0.006
Occupational burnout	0.145	0.025	1.156	1.101	1.214	< 0.001
Years of service	0.033	0.016	1.033	1.000	1.067	0.047

Decision Tree Model: The results showed that the tree grew to a depth of 5 layers, comprising 19 nodes in total, with 10 terminal nodes. The model identified six key explanatory variables: work–family conflict, occupational stress, burnout, nursing practice environment, years of work, and sleep disorders. Work–family conflict served as the root node, indicating it had the strongest association with WPV among emergency nurses. For nurses with high work–family conflict, occupational stress and burnout were the most significant associated factors of WPV (located in the second layer of the model), followed by years of experience, work environment, and sleep disorders (located in the third, fourth, and fifth layers, respectively). Notably, emergency nurses with high work–family conflict, high occupational stress, and high burnout showed the highest proportion of nurses reporting WPV experiences. The ranking of the importance of each variable in the decision tree model is shown in Table 4.

**TABLE 4:** Ranking of variable importance in the decision tree model for WPV among emergency department nurses.

Variable	Importance	Ranking
Work‐to‐family conflict	59.487	1
Occupational stress	40.174	2
Occupational burnout	29.555	3
Work environment	9.567	4
Years of service	7.118	5
Sleep disorders	5.574	6

**FIGURE 2:**
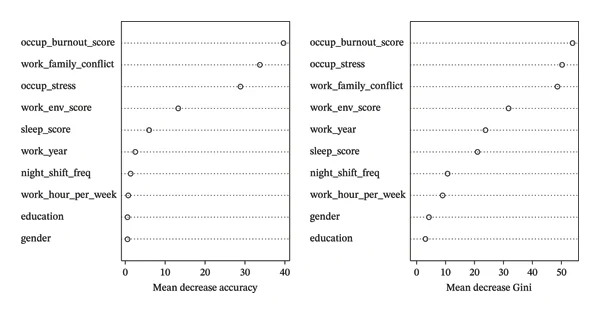
Mean decrease accuracy and mean decrease Gini of random forest.

**FIGURE 3:**
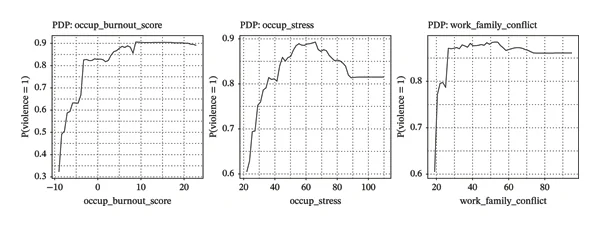
Dependence plots for the top‐ranked predictors to random forest.

**FIGURE 4:**
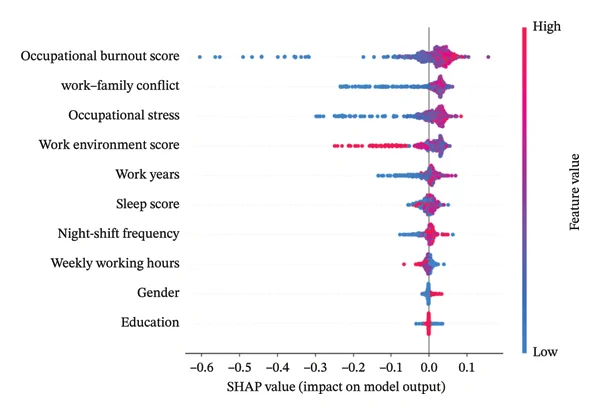
SHAP value analysis of random forest.

Random Forest Model: The importance of these associated factors was ranked by comparing their mean decrease in the Gini index, which explains each variable’s contribution to assessing WPV among emergency department nurses. A larger Gini coefficient indicates a greater contribution of the associated factors variable to the model’s classification decisions and a greater contribution to classification performance contribution. The results showed that burnout was the most important factor associated with WPV experiences, with a Gini coefficient of 53.778, ranking first. Occupational stress and work–family conflict were the next most important associated factors, with Gini coefficients of 50.213 and 48.553, respectively. The nursing practice environment, years of work experience, presence of sleep disorders, monthly income, and night shift work also contributed to the model’s classification to varying degrees, as detailed in Table 5. Variable importance analysis, partial dependence plots, and SHAP value analysis of random forest are shown in Figures 2, 3, and 4.

**TABLE 5:** Ranking of variable importance in the random forest model of WPV for emergency department nurses.

Variable	Mean decrease Gini	Ranking
Occupational burnout	53.778	1
Occupational stress	50.213	2
Work‐to‐family conflict	48.553	3
Work environment	31.768	4
Years of service	23.809	5
sleep disorders	21.010	6

**FIGURE 5:**
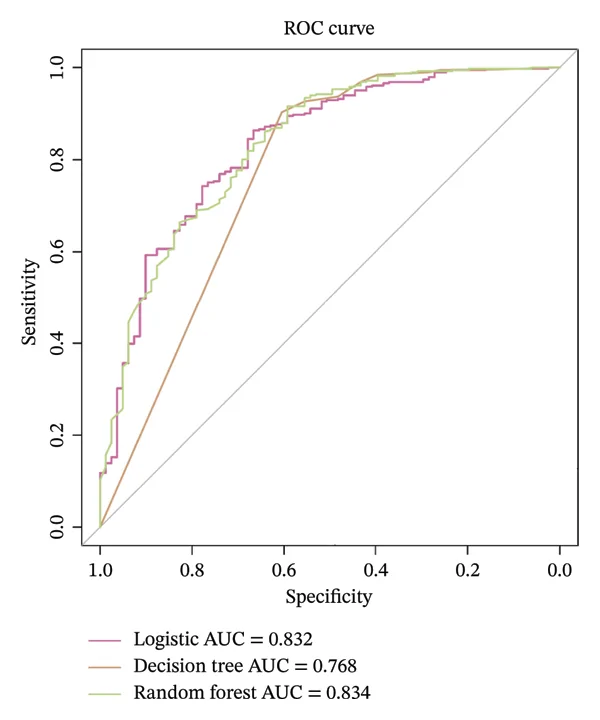
Receiver operating characteristic curves of the three machine learning models for workplace violence among emergency nurses.

The classification performance and discriminative capabilities of the three models were rigorously evaluated on the independent testing dataset. As illustrated by the ROC curves in Figure 5, the random forest model achieved the highest (AUC = 0.834, 95% CI: 0.783–0.885), closely followed by the logistic regression model (AUC = 0.832, 95% CI: 0.781–0.883), while the decision tree model exhibited the lowest discrimination (AUC = 0.768, 95% CI: 0.711–0.826).

**FIGURE 6:**
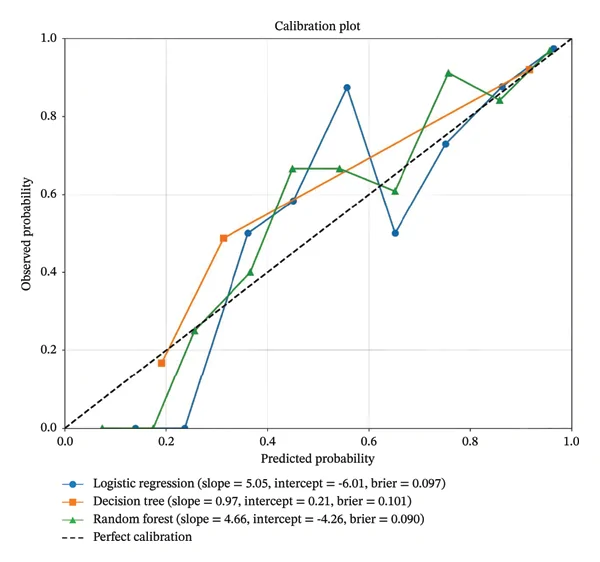
The calibration plots of the three models.

The comprehensive performance metrics are summarized in Table 6. Due to the high baseline prevalence of WPV in the study population, all three models consistently maintained high positive predictive values (PPV > 0.9000). Random forest achieved the highest sensitivity (0.916), F1‐score (0.915), and overall accuracy (0.859), whereas logistic regression showed the highest specificity (0.667) and balanced accuracy (0.765). Logistic regression and random forest showed nearly identical observed discrimination, with AUC values of 0.832 and 0.834, respectively. The relatively low specificity and negative predictive values across the models indicated more limited performance in identifying nurses without WPV. The DeLong test was performed to compare the AUC of logistic regression (0.834) and random forest (0.832). The difference in AUC was not statistically significant (ΔAUC = −0.017, *p* = 0.377; 95% CI: −0.053–0.020), indicating comparable discriminative ability between the two models. Model calibration was further assessed using the Brier score, shown in Figure 6. The Brier scores for the random forest, logistic regression, and decision tree models were 0.090, 0.97, and 0.101, respectively. Confusion matrices were added for all three models. For the random forest model, using the Youden‐optimal threshold of 0.707, the model correctly classified 349 positive cases and 48 negative cases in the test set, while 32 positive cases and 33 negative cases were misclassified, details shown in Table S3.

**TABLE 6:** Comparative performance of the three machine learning models (test set).

Model	Threshold	AUC	Sensitivity	Specificity	PPV	NPV	F1‐score	Bal. Acc.	Acc.	Calibrate slope	Calibrate intercept	Brier score	ECE
Logistic regression	0.7853	0.832	0.864	0.667	0.924	0.509	0.893	0.765	0.829	5.05	−6.01	0.0973	0.0396
Decision tree	0.9130	0.768	0.903	0.605	0.915	0.570	0.909	0.754	0.851	0.97	0.21	0.1010	0.0251
Random forest	0.7070	0.834	0.916	0.593	0.914	0.600	0.915	0.754	0.859	4.66	−4.26	0.0899	0.0432

*Note:* AUC: receiver operating characteristic curve, Bal. Acc.: balanced accuracy, Acc: accuracy.

Abbreviations: ECE, expected calibration error; NPV, negative predictive value; PPV, positive predictive value.

## 4. Discussion

Among the three models evaluated, the random forest model achieved the best overall classification performance, demonstrating the highest sensitivity, specificity, F1‐score, balanced accuracy, and the lowest Brier score. However, its discriminative ability was only marginally higher than that of logistic regression (AUC: 0.834 vs. 0.832), suggesting that a conventional regression approach adequately captured the associations between the identified characteristics and WPV experiences in the present dataset [26]. Logistic regression remains advantageous because of its transparency and interpretability, whereas random forest demonstrated greater robustness across multiple evaluation metrics [27]. Although the decision tree model exhibited lower performance, its transparent decision rules may still facilitate practical implementation when interpretability is prioritized. Therefore, it may still be useful as a practical screening approach when model interpretability is prioritized [28]. Overall, these findings suggest that while random forest achieved the best overall performance, model selection should balance classification performance with interpretability according to the intended application.

Work–family conflict, occupational burnout, occupational stress, nursing practice environment, and sleep disorders were consistently ranked among the most influential variables across the models. Rather than representing isolated individual characteristics, these variables may reflect common underlying organizational and psychosocial burdens within emergency nursing environments [29]. Emergency departments are characterized by high workloads, time pressure, overcrowding, frequent interruptions, and emotionally demanding interactions [30]. Such conditions may contribute simultaneously to burnout, occupational stress, work–family conflict, sleep disturbances, and exposure to WPV [31]. Recent studies have also emphasized the role of workload, staffing adequacy, leadership support, and organizational resources in shaping nurses’ experiences of WPV [32]. The present findings therefore support the view that WPV should be considered within a broader organizational and occupational context [33]. Therefore, interventions targeting organizational conditions may simultaneously improve multiple psychosocial indicators rather than addressing each factor separately.

Work–family conflict was one of the highest‐ranked variables across the models. Emergency nurses frequently face competing demands from professional and family responsibilities because of shift work, long working hours, and irregular schedules [34]. Previous studies have linked work–family conflict to emotional exhaustion, reduced job satisfaction, and psychological distress among nurses [35]. The association observed in the present study suggests that difficulties in balancing work and family responsibilities may also be related to WPV experiences.

Burnout and occupational stress were also strongly associated with WPV. Similar findings have been reported in previous studies of emergency nurses [36]. Because of the cross‐sectional design, the direction of these relationships remains uncertain. Burnout and stress may precede, accompany, or result from violence exposure [37]. Regardless of the direction of the association, these findings indicate that WPV occurs within a broader context of psychological strain and demanding working conditions [38].

The nursing practice environment was another important factor associated with WPV. Supportive practice environments are generally characterized by adequate staffing, effective teamwork, managerial support, and organizational resources [39]. These factors may reduce workplace tensions and improve nurses’ ability to manage difficult interactions. Sleep disorders were also associated with WPV across the models [40]. Given the high prevalence of shift work in emergency departments, sleep disturbances may reflect inadequate recovery from demanding work schedules and ongoing occupational strain. Both findings underscore the importance of considering organizational conditions alongside individual well‐being in WPV prevention efforts.

Model interpretability is an important consideration when selecting analytical approaches for clinical and organizational applications. Logistic regression provides transparent estimates of associations through odds ratios [41], whereas random forest operates as a more complex model with limited transparency [16] Given the comparable discrimination performance observed in this study, model selection should consider not only classification performance but also interpretability and practical applicability.

Therefore, in datasets with relatively simple relationships and moderate dimensionality, conventional logistic regression may provide sufficient performance while retaining greater interpretability. Machine learning methods may become more advantageous when larger datasets with more complex nonlinear interactions are available.

All the variables in this study were measured at a single time point. Therefore, the identified factors should be regarded as characteristics related to the WPV experience, rather than predictors of future WPV events. Thus, longitudinal studies need to be conducted in the future to clarify the temporal and spatial relationships among WPV, psychological and social factors, and organizational conditions. These relevant factors can be prioritized through machine learning. WPV is influenced by healthcare system characteristics, organizational culture, staffing models, and sociocultural factors. Therefore, caution is needed when generalizing these findings to other countries and healthcare settings.

### 4.1. Limitations

Several limitations should be acknowledged. First, the cross‐sectional design limits the ability to determine the direction of the relationships between WPV and the associated factors identified in this study. Second, all variables were measured using self‐reported questionnaires, which may be influenced by recall bias. Third, different forms and sources of WPV, including physical and psychological violence as well as patient‐to‐staff and staff‐to‐staff violence, were combined into a single outcome. As these experiences may have different underlying mechanisms, the findings should be interpreted with caution. Fourth, the absence of hospital‐level identifiers and a defined sampling denominator precluded estimation of hospital participation rates, regional response rates, and within‐hospital clustering. Although participants were recruited from multiple regions across China, only internal validation was performed. Therefore, external validation in independent healthcare settings is needed before the models can be generalized more broadly. Finally, because all variables and WPV experiences were measured at the same time, the models assess cross‐sectional associations and classification of current WPV status rather than prediction of future WPV events. Future studies should also examine different forms and sources of WPV separately.

### 4.2. Implications for Practice

The findings suggest that WPV prevention should extend beyond incident management and focus on broader psychosocial and organizational conditions. Work–family conflict, occupational burnout, occupational stress, sleep disorders, and the nursing practice environment were consistently associated with WPV experiences across the models and may serve as useful indicators for workplace monitoring.

The identified factors may be incorporated into routine workforce assessments to help managers better understand the psychosocial conditions of emergency nurses and guide targeted organizational interventions. The variable importance rankings generated by the machine learning models may also help prioritize intervention targets and support resource allocation.

Operationally, a brief questionnaire could be administered every 3 to 6 months at the unit level for monitoring purposes. When elevated levels of burnout, occupational stress, or work–family conflict are observed, managers may consider organizational strategies such as optimizing staffing levels, improving shift scheduling practices, strengthening leadership support, enhancing WPV prevention training, and providing access to psychological support services. Nurses identified as having multiple adverse psychosocial indicators could receive individualized follow‐up assessments, targeted psychological support, workload adjustments, or mentoring programs according to local organizational policies. Such measures may contribute to healthier work environments and support broader WPV prevention efforts.

## 5. Conclusion

This study compared the performance of logistic regression, decision tree, and random forest models in classifying WPV experiences among emergency nurses. All three models demonstrated acceptable discrimination performance, with logistic regression and random forest achieving similar performance and higher AUC values than the decision tree model. Across models, work–family conflict, occupational burnout, occupational stress, nursing practice environment, and sleep disorders were consistently associated with WPV experiences. These findings highlight the potential importance of psychosocial and organizational factors in understanding WPV among emergency nurses.

## Author Contributions

Lin Lan: supervision, validation, data curation, formal analysis, writing–original draft, and writing–review and editing.

Min Dai: investigation, methodology, supervision, and writing–review and editing.

Yilong Chen: data curation, formal analysis, and project administration.

Hao Zhang: investigation, formal analysis, project administration, and writing–review and editing.

Luying Zhong: investigation, software, data curation, and project administration.

Xiaoli Chen: conceptualization, project administration, resources, validation, writing–original draft, and writing–review and editing.

## Funding

The author(s) declare that no financial support was received for the research, authorship, and/or publication of this article.

## Disclosure

We hereby confirm that the research presented in this manuscript represents the original work of the listed authors. No third‐party services or unacknowledged individuals participated in any part of this work. All individuals who contributed substantively to this study have been included as co‐authors. The final manuscript has been reviewed and approved by all named authors, and they take full responsibility for its content.

## Ethics Statement

This study was approved by the Ethics Review Committee of West China Hospital, Sichuan University (Approval No. 2024.309), on February 20, 2024. All participants provided informed consent and voluntarily participated in the study.

## Conflicts of Interest

The authors declare no conflicts of interest.

## Supporting Information

Additional supporting information can be found online in the Supporting Information section.

## Supporting information


**Supporting Information 1** Table S1: Univariate analysis of WPV against nurses in the emergency department. Table S2: Variance inflation factor (VIF) values for predictors in the logistic regression model. Table S3: The confusion matrices for three models.

**Supporting Information 2** STROBE Statement—Checklist of items that should be included in reports of cross‐sectional studies.

## Data Availability

The data that support the findings of this study are available on request from the corresponding author.

## References

[1] Ma P. F. and Thomas J. , Workplace Violence in Healthcare, 2025, https://pubmed.ncbi.nlm.nih.gov/37276305/. 37276305

[2] Hou Y. , Corbally M. , and Timmins F. , Violence Against Nurses by Patients and Visitors in the Emergency Department: A Concept Analysis, Journal of Nursing Management. (2022) 30, no. 6, 1688–1699, 10.1111/jonm.13721. 35700325 PMC9795924

[3] Boles J. M. , Maccarone D. , Brown B. et al., Nurse, Provider, and Emergency Department Technician: Perceptions and Experiences of Violence and Aggression in the Emergency Department, Journal of Emergency Nursing. (2023) 49, no. 3, 431–440, 10.1016/j.jen.2022.07.008. 36180265

[4] Öztaş İ. , Yava A. , and Koyuncu A. , Exposure of Emergency Nurses to Workplace Violence and Their Coping Strategies: A Cross-Sectional Design, Journal of Emergency Nursing. (2023) 49, no. 3, 441–449, 10.1016/j.jen.2022.09.002. 36307253

[5] Li N. , Zhang L. , Xiao G. , Chen J. , and Lu Q. , The Relationship Between Workplace Violence, Job Satisfaction and Turnover Intention in Emergency Nurses, International Emergency Nursing. (2019) 45, 50–55, 10.1016/j.ienj.2019.02.001. 30797732

[6] Liu J. , Zheng J. , Liu K. et al., Workplace Violence Against Nurses, Job Satisfaction, Burnout, and Patient Safety in Chinese Hospitals, Nursing Outlook. (2019) 67, no. 5, 558–566, 10.1016/j.outlook.2019.04.006. 31202444

[7] Ajuwa M. P. , Veyrier C. A. , Cousin Cabrolier L. et al., Workplace Violence Against Female Healthcare Workers: A Systematic Review and Meta-Analysis, BMJ Open. (2024) 14, no. 8, 10.1136/bmjopen-2023-079396. PMC11369783 39209501

[8] Kumari A. , Sarkar S. , Ranjan P. et al., Interventions for Workplace Violence Against Health-Care Professionals: A Systematic Review, Work. (2022) 73, no. 2, 415–427, 10.3233/WOR-210046. 35431213

[9] Jia H. , Fang H. , Chen R. et al., Workplace Violence Against Healthcare Professionals in a Multiethnic Area: A Cross-Sectional Study in Southwest China, BMJ Open. (2020) 10, no. 9, 10.1136/bmjopen-2020-037464. PMC7482505 32907902

[10] Kumari A. , Kaur T. , Ranjan P. , Chopra S. , Sarkar S. , and Baitha U. , Workplace Violence Against Doctors: Characteristics, Risk Factors, and Mitigation Strategies, Journal of Postgraduate Medicine. (2020) 66, no. 3, 149–154, 10.4103/jpgm.JPGM_96_20. 32675451 PMC7542052

[11] Al-Qadi M. M. , Workplace Violence in Nursing: A Concept Analysis, Journal of Occupational Health. (2021) 63, no. 1, 10.1002/1348-9585.12226. PMC8103077 33960074

[12] Choi R. Y. , Coyner A. S. , Kalpathy-Cramer J. , Chiang M. F. , and Campbell J. P. , Introduction to Machine Learning, Neural Networks, and Deep Learning, Translational Vision Science & Technology. (2020) 9, no. 2, 10.1167/tvst.9.2.14. PMC7347027 32704420

[13] Havaei F. , Adhami N. , Tang X. et al., Workplace Predictors of Violence Against Nurses Using Machine Learning Techniques: A Cross-Sectional Study Utilizing the National Standard of Psychological Workplace Health and Safety, Healthcare. (2023) 11, no. 7, 10.3390/healthcare11071008. PMC10094471 37046935

[14] Luo L. , Wu Y. , Li S. , Li F. , Wang X. , and Wei X. , Identifying the Most Critical Predictors of Workplace Violence Experienced by Junior Nurses: An Interpretable Machine Learning Perspective, Journal of Nursing Management. (2025) 2025, no. 1, 10.1155/jonm/5578698. PMC11981708 40223873

[15] Karalis G. , Decision Trees and Applications, Advances in Experimental Medicine and Biology. (2020) 1194, 239–242, 10.1007/978-3-030-32622-7_21. 32468539

[16] Hu J. and Szymczak S. , A Review on Longitudinal Data Analysis With Random Forest, Briefings in Bioinformatics. (2023) 24, no. 2, 10.1093/bib/bbad002. PMC10025446 36653905

[17] Yuan G. P. , Jiang Q. , Sun K. et al., Research Progress on Post-Traumatic Stress Disorder and Post-Traumatic Growth Among Emergency Department Nurses Exposed to Workplace Violence, Nursing and Rehabilitation. (2025) 24, no. 7, 87–90.

[18] Hesketh K. L. , Duncan S. M. , Estabrooks C. A. et al., Workplace Violence in Alberta and British Columbia Hospitals, Health Policy. (2003) 63, no. 3, 311–321, 10.1016/s0168-8510(02)00142-2. 12595130

[19] Zhang Y. Q. , Revision and Application of the Simplified Work-Family Behavior Role Conflict Scale, 2023, Guizhou Normal University, 10.7666/d.D03086648.

[20] Clark M. A. , Early R. J. , Baltes B. B. , and Krenn D. , Work-Family Behavioral Role Conflict: Scale Development and Validation, Journal of Business and Psychology. (2019) 34, no. 1, 39–53.

[21] Niedhammer I. , Tek M. L. , Starke D. , and Siegrist J. , Effort-Reward Imbalance Model and Self-Reported Health: Cross-Sectional and Prospective Findings From the GAZEL Cohort, Social Science & Medicine. (2004) 58, no. 8, 1531–1541, 10.1016/S0277-9536(03)00346-0. 14759696

[22] Maslach C. and Leiter M. P. , Understanding the Burnout Experience: Recent Research and Its Implications for Psychiatry, World Psychiatry. (2016) 15, no. 2, 103–111, 10.1002/wps.20311. 27265691 PMC4911781

[23] Zhu W. , Lou X. P. , and Wang Z. M. , Evaluation of the Reliability and Validity of the Maslash Burnout Inventory-General Survey in Nursing Staff, Chinese Journal of Behavioral Medical Science. (2007) 16, no. 9, 849–851, 10.3760/cma.j.issn.1674-6554.2007.09.031.

[24] Ye Z. H. , Practice of Hospital Nursing Work Environment Construction, Chinese Nursing Management. (2013) 13, no. 2, p1–p3, 10.3969/j.issn.1672-1756.2013.02.001.

[25] Fabbri M. , Beracci A. , Martoni M. , Meneo D. , Tonetti L. , and Natale V. , Measuring Subjective Sleep Quality: A Review, International Journal of Environmental Research and Public Health. (2021) 18, no. 3, 10.3390/ijerph18031082. PMC7908437 33530453

[26] Zuo F. , Su J. , Li Y. et al., Development and Validation of a Machine Learning-Based Model for Predicting Intraoperative Blood Loss During Burn Surgery, Surgery. (August 2025) 184, 10.1016/j.surg.2025.109445. 40446607

[27] Peng Y. , Zhang C. , and Zhou B. , A Cross-Sectional Study Comparing Machine Learning and Logistic Regression Techniques for Predicting Osteoporosis in a Group at High Risk of Cardiovascular Disease Among Old Adults, BMC Geriatrics. (March 2025) 25, no. 1, 10.1186/s12877-025-05840-w. PMC11954202 40158079

[28] Cesare M. , Nurchis M. C. , Damiani G. , and Cocchieri A. , Ranking Nursing Diagnoses by Predictive Relevance for Intensive Care Unit Transfer Risk in Adult and Pediatric Patients: A Machine Learning Approach With Random Forest, Healthcare. (2025) 13, no. 11, 10.3390/healthcare13111339. PMC12154488 40508953

[29] Cesare M. , Di Nitto M. , Iovino P. et al., Prevalence and Determinants of Workplace Violence Against Nurses in the Italian Home Care Settings: A Cross-Sectional Multicentre Study, Journal of Clinical Nursing. (2026) 35, no. 5, 2288–2298, 10.1111/jocn.70007. 40596785 PMC13068183

[30] Lee H. , Yun H. , Choi M. , and Kim H. , Predicting Workplace Violence in the Emergency Department Based on Electronic Health Record Data, Journal of Emergency Nursing. (2023) 49, no. 3, 415–424, 10.1016/j.jen.2023.01.010. 36925384

[31] Bildik B. , Atis S. E. , Cekmen B. , and Dorter M. , Do We Feel Safe and Confident About Workplace Violence in the Emergency Departments?, American Journal of Emergency Medicine. (2022) 59, 9–14, 10.1016/j.ajem.2022.06.040. 35772226

[32] Tsukamoto S. A. S. , Galdino M. J. Q. , Barreto M. F. C. , and Martins J. T. , Burnout Syndrome and Workplace Violence Among Nursing Staff: A Cross-Sectional Study, Sao Paulo Medical Journal. (2022) 140, no. 1, 101–107, 10.1590/1516-3180.2021.0068.R1.31052021. 34932780 PMC9623842

[33] Zhang H. , Zhou J. , Zhong L. , Zhu L. , and Chen X. , Relationship Between Workplace Violence and Occupational Health in Emergency Nurses: The Mediating Role of Dyssomnia, Nursing in Critical Care. (2025) 30, no. 2, 10.1111/nicc.70008. 40075212

[34] Tutan A. and Kökalan Ö. , The Mediation Role of Work-Family Conflict in the Effect of Workplace Violence on Job Satisfaction and Intention to Leave: A Study on Health Care Workers in Turkey, Frontiers in Psychology. (2024) 15, 10.3389/fpsyg.2024.1322503. PMC11033917 38650903

[35] Diao D. , Chen X. , Zhong L. , Zhang H. , and Zhang J. , Sex Differences in Burnout and Work-Family Conflict Among Chinese Emergency Nurses: A Cross-Sectional Study, Frontiers in Public Health. (2024) 12, 10.3389/fpubh.2024.1492662. PMC11659251 39712298

[36] Lan L. , Chen X. , Zhang H. , Zhong L. , and Ye L. , Characterizing Potential Subtypes and Influencing Factors of Burnout in Emergency Department Nurses by Latent Profile Analysis, Frontiers in Public Health. (2025) 13, 10.3389/fpubh.2025.1654398. PMC12507872 41080872

[37] Amiri S. , Mahmood N. , Mustafa H. , Javaid S. F. , and Khan M. A. , Occupational Risk Factors for Burnout Syndrome Among Healthcare Professionals: A Global Systematic Review and Meta-Analysis, International Journal of Environmental Research and Public Health. (2024) 21, no. 12, 10.3390/ijerph21121583. PMC11675210 39767426

[38] Somani R. , Muntaner C. , Hillan E. , Velonis A. J. , and Smith P. , A Systematic Review: Effectiveness of Interventions to De-Escalate Workplace Violence Against Nurses in Healthcare Settings, Safety and Health at Work. (2021) 12, no. 3, 289–295, 10.1016/j.shaw.2021.04.004. 34527388 PMC8430427

[39] Lake E. T. , Sanders J. , Duan R. , Riman K. A. , Schoenauer K. M. , and Chen Y. , A Meta-Analysis of the Associations Between the Nurse Work Environment in Hospitals and 4 Sets of Outcomes, Medical Care. (2019) 57, no. 5, 353–361, 10.1097/MLR.0000000000001109. 30908381 PMC6615025

[40] Qiu D. , Yu Y. , Li R. Q. , Li Y. L. , and Xiao S. Y. , Prevalence of Sleep Disturbances in Chinese Healthcare Professionals: A Systematic Review and Meta-Analysis, Sleep Medicine. (2020) 67, 258–266, 10.1016/j.sleep.2019.01.047. 31040078

[41] Wang Q. Q. , Overview of Logistic Regression Model Analysis and Application, Zhonghua Yufang Yixue Zazhi. (2019) 53, no. 9, 955–960, 10.3760/cma.j.issn.0253-9624.2019.09.018. 31474082

