# Supplementary material for: Machine Learning Models for Identifying Factors Associated With Workplace Violence Among Emergency Nurses: A Comparative Study
Source: Emerg Med Int. 2026 Aug 19;2026:9275645. doi: 10.1155/emmi/9275645 (PMC13487808; doi:10.1155/emmi/9275645)
Supplement: Supplementary file 1 [file EMMI-2026-9275645-s001.docx]

Table S1 Univariate Analysis of WPV Against Nurses in the Emergency Department

| Variable | Category | WPV | |  |  |
| --- | --- | --- | --- | --- | --- |
|  |  | No | Yes | chi-square value/Finsher value | P |
| Age（years） | 20~29 | 124 | 458.0 | 28.847 | < 0.001 |
|  | 30~39 | 83 | 651.0 |  |  |
|  | 40~49 | 18 | 165.0 |  |  |
|  | ≥50 | 6 | 35.0 |  |  |
| Gender | female | 182 | 1029.0 | 0.004 | 1.000 |
|  | male | 49 | 280.0 |  |  |
| Marital status | unmarried | 112 | 448.0 | 16.644 | < 0.001 |
|  | Married | 119 | 861.0 |  |  |
| Fertility status | nonparous | 128 | 582.0 | 9.039 | 0.003 |
|  | fertilized | 103 | 727.0 |  |  |
| Education | Junior college | 45 | 141.0 | 13.216 | < 0.001 |
|  | Bachelor’s degree and above | 186 | 1168.0 |  |  |
| Job title | Primary | 165 | 742.0 | 19.625 | < 0.001 |
|  | Intermediate | 63 | 511.0 |  |  |
|  | Senior | 3 | 56.0 |  |  |
| Years of service（years） | ≤2 | 65 | 177.0 | 33.429 | < 0.001 |
|  | 3~10 | 108 | 634.0 |  |  |
|  | 11~20 | 41 | 380.0 |  |  |
|  | ≥21 | 17 | 118.0 |  |  |
| Total weekly working hours (h) | ≤40 | 92 | 485.0 | 0.681 | 0.878 |
|  | 41~48 | 113 | 667.0 |  |  |
|  | 49~58 | 17 | 106.0 |  |  |
|  | ≥59 | 9 | 51.0 |  |  |
| Night shifts | Yes | 196 | 1163.0 | 2.653 | 0.103 |
|  | No | 35 | 146.0 |  |  |
| Night shifts（times/month） | 0 | 35 | 146.0 | 4.473 | 0.215 |
|  | 1~4 | 36 | 203.0 |  |  |
|  | 5~8 | 101 | 558.0 |  |  |
|  | ＞8 | 59 | 402.0 |  |  |
| Monthly income (CNY)（yuan） | ＜4000 | 13 | 44.0 | 17.443 | < 0.001 |
|  | 4000~5999 | 37 | 112.0 |  |  |
|  | 6000~7999 | 35 | 215.0 |  |  |
|  | 8000~9999 | 58 | 310.0 |  |  |
|  | ≥10000 | 88 | 628.0 |  |  |
| Smoking | No | 220 | 1233.0 | 0.230 | 0.632 |
|  | Yes | 11 | 76.0 |  |  |
| Drinking | No | 207 | 1148.0 | 0.509 | 0.476 |
|  | Yes | 24 | 161.0 |  |  |
| Occupational Burnout | ＜1.5 | 42 | 36.0 | 96.998 | < 0.001 |
|  | 1.5~3.5 | 28 | 52.0 |  |  |
|  | ≥3.5 | 161 | 1221.0 |  |  |
| Sleep disorders | Yes | 113 | 800.0 | 11.603 | < 0.001 |
|  | No | 118 | 509.0 |  |  |
| Occupational stress | High pressure | 16.000 | 387.0 | 69.495 | 0.000 |
|  | Balance | 11.000 | 94.0 |  |  |
|  | Low pressure | 204.000 | 828.0 |  |  |

Table S2 Variance inflation factor (VIF) values for predictors in the logistic regression model

| Variable | Variance inflation factor (VIF) |
| --- | --- |
| Work-to-family conflict | 1.92 |
| Occupational stress | 2.83 |
| Occupational Burnout | 1.87 |
| Years of service | 2.87 |
| Work environment | 1.38 |
| Sleep disorders | 1.29 |

Table S3 The confusion matrices for three models

| **Model** | **Threshold** | **TP** | **TN** | **FP** | **FN** |
| --- | --- | --- | --- | --- | --- |
| Logistic Regression | 0.7853 | 329 | 54 | 52 | 27 |
| Decision Tree | 0.9130 | 344 | 49 | 37 | 32 |
| Random Forest | 0.7070 | 349 | 48 | 32 | 33 |
